# A Laser-Based On-Machine Measuring System for Profile Accuracy of Double-Headed Screw Rotor

**DOI:** 10.3390/s19235059

**Published:** 2019-11-20

**Authors:** Zhixu Dong, Fangsu Xu, Xingwei Sun, Weijun Liu

**Affiliations:** School of Mechanical Engineering, Shenyang University of Technology, Shenyang 110870, China; dong_zhixu@sut.edu.cn (Z.D.); xu_fangs@126.com (F.X.); wjliu@sia.cn (W.L.)

**Keywords:** screw rotor, laser displacement sensor, optical inspection, data processing

## Abstract

Great length, large weight and other factors may cause difficulty in measuring the profile accuracy of the double-headed screw rotor. To solve this problem, an on-machine measuring system based on a laser-displacement sensor (LDS) was designed and implemented in this paper by taking an LXK100 four-axis whirlwind milling machine as the carrier. To improve the measurement accuracy of the system, the generalized variable-structural-element morphological method, polynomial interpolation algorithm and ellipse fitting method were first combined to realize the rapid subpixel centroid extraction from a noise-containing spot image, thus improving the data acquisition accuracy of the LDS, and then the hybrid method was experimentally verified. Next, a wavelet threshold function with high-order differentiability and adaptive wavelet coefficient contractility was constructed based on the hyperbolic tangent function, so as to inhibit the disturbance from random errors and preserve real profile information, and this method was simulated and verified. Subsequently, a smoothing algorithm for point cloud data was proposed based on the Lagrange multiplier method to avoid the defect of the piecewise curve-fitting method, that is, function continuity and differentiability could not be satisfied at piecewise points. Finally, the profile accuracy was calculated in real time according to the data reconstruction result and the machining quality was judged. The measurement experiment of the double-headed screw rotor indicates that the proposed on-machine measuring system can complete the profile accuracy measurement for a screw pitch within 39.7 s with measurement accuracy reaching ±8 μm, and the measurement uncertainties of the major axis, minor axis and screw pitch are 0.72 μm, 0.69 μm and 1.24 μm, respectively. Therefore, the measurement accuracy and efficiency are both remarkably improved.

## 1. Introduction

Complex spiral mechanical equipment has been applied extensively to such fields as petrochemical engineering, biomedicine, environmental water treatment and papermaking, with the rapid development of manufacturing technologies for spiral curved surfaces. Among them, the spiral dynamic mechanical screw pump and screw motor are the most common. As a critical power-generation part commonly used in the aforementioned equipment, the double-headed screw rotor has a direct bearing on the mechanical properties and service life of the product with its profile accuracy [1]. It is well known that measurement is an effective method of improving the machining and manufacturing accuracy, and the current profile accuracy measuring methods for double-headed screw rotors are all contact-type, divided into two types: The one type is to manually measure the profile accuracy with a single parameter measuring instrument such as a micrometer. Its measurement accuracy and efficiency are both low although this method can realize on-machine measurement. The other type refers to automatic measurement of the profile accuracy with a coordinate measuring machine (CMM). Despite its high measurement accuracy, this method consumes a lot of auxiliary time and increases the positioning error in off-machine measurement and, meanwhile, cannot guarantee the post-correction accuracy of the non-conforming screw. In recent years, it has become an important means to improve the profile manufacturing accuracy and efficiency of many mechanical parts by using non-contact scanning measurement technology to construct an on-machine measuring system that integrates “machining-measurement-compensation” [2]. Among numerous non-contact sensors, the laser-displacement sensor (LDS) has been widely used owing to its advantages including high accuracy, large range, high speed and favorable stability. Li et al. used the combined measurement method of multiple LDSs to construct an on-machine quality control system for an automobile engine cylinder [3]. Alarm et al. designed and implemented an LDS-based on-machine measuring device for paper quality [4]. Brosed et al. combined the LDS with a six-axis robot to realize the on-machine measurement for products with complex geometrical shapes [5]. However, in the studies above, the accuracy of a commercialized LDS is considered equal to that of the whole measuring system, and there is a lack of effective processing methods for LDS signals. Consequently, the measurement results are unsatisfactory. Korosec stated that the final accuracy of the aforementioned industrial applications relies on the accuracy of the signal collected by sensors and the subsequent signal reconstruction [6].

Many factors that affect the data acquisition accuracy of the LDS are generally divided into four aspects: inherent characteristics of the measuring system, measuring environment, physical properties of the measured surface, and the extraction method for spot centroids. As for inherent characteristics, many scholars have investigated three major problems: non-linearity of the laser triangulation model [7], disturbance of laser beam [8] and positional relation of LDS system elements [9], and they have obtained good effects, thus improving the data acquisition accuracy of the LDS to a certain degree. In terms of measuring environment, scholars have studied the effects of ambient light change [10], ambient temperature and humidity [11], vibration [12] and non-uniform refractive index field in the environment [13] on the measurement accuracy of the LDS, respectively. In addition, they have mastered the influence laws of different environmental factors and raised the corresponding compensation methods. When it comes to the physical properties of the measured surface, scholars have deeply probed the effects of color [14], luminance [15] and curvature [16] of the measured surface as well as dip angle of the measurement point [17] on the measurement accuracy of the LDS, and the results they achieved have notably improved the data acquisition accuracy of the LDS in complex surfaces. Through the aforementioned studies, any influence factor can lead to the centroid skewing phenomenon in the spot image, which finally results in the generation of measuring errors. Therefore, it is of great importance to improve the data acquisition accuracy of the LDS by realizing rapid and accurate centroid extraction from the spot image. The grayscale centroid extraction method has been widely applied to spot centroid extraction because of its simple operation and high calculation speed, but it is particularly sensitive to noise, resulting in low accuracy [18]. Wu, Selami and Yin et al. used the Hessian matrix method, an improved Gaussian fitting method, and the cubic Hermite spline interpolation method to perform grayscale centroid extraction, respectively. Although the above methods have improved the extraction accuracy of spot centroids to a certain extent, they cannot improve the robustness of the algorithm yet [19,20,21]. To solve this problem, Li et al. improved the poor robustness by Steger method. This method improved the accuracy and robustness of the centroid extraction method based on a differential operation, but it is not ideal for processing noise-containing spot images [22]. Even though Liu, He and Jiang et al. improved the Steger method through a graphics processing unit (GPU), cross-structured light and field programmable gate array (FPGA), which enhanced the noise jamming resistance of the Steger method, they could not realize accurate centroid extraction from spot images with outliers and large interference [23,24,25].

The data reconstruction of the LDS mainly includes denoising and fitting. As for the former, the data acquired through the LDS in the measuring process certainly contain random noises under the influence of the aforementioned factors, so denoising is necessary. It is well known that simultaneous realization of noise inhibition and detail preservation has always been a problem faced in the image-denoising field. Therefore, it has been proved that the discrete wavelet transform (DWT) is a key technology to eliminate the noise by virtue of its high effectiveness. Nevertheless, the construction of threshold function directly affects the data-processing quality of DWT denoising methods. Donoho and Johnstone put forward the hard and soft thresholding functions, which were widely applied to the field of DWT denoising [26]. However, the two methods also have some drawbacks: the disadvantages of the hard denoising function and soft denoising function are the continuity and constant deviation problems, respectively. To solve the above problems, Yi, Xu and Gao et al. established different threshold functions, which overcame disadvantages of the traditional soft and hard threshold functions while guaranteeing good denoising effects [27,28,29]. However, the above improved methods do not have high-order differentiability, so real data information will be eliminated while the noise jamming is removed. Moreover, the stronger the denoising capacity, the more serious the elimination of real data will be, thus causing data distortion after the reconstruction and influencing the final measurement accuracy. In the aspect of fitting, the least square method has been extensively used for piecewise fitting by virtue of its high fitting efficiency and simple operation [30]. Nevertheless, in view of the fact that the continuity and differentiability of the function cannot be satisfied at piecewise points, the data fitting precision is poor, thus affecting the final measurement accuracy.

To solve the above problems, in this paper we designed and implemented an LDS-based on-machine measuring system for the profile accuracy of a double-headed screw rotor, taking the LXK100 four-axis whirlwind milling machine as the carrier. As for the LDS data acquisition, a new-type centroid extraction method that resists noise jamming was put forward. In this method, the generalized variable-structural-element morphological method and polynomial interpolation algorithm were first used to conduct rapid subpixel positioning for effective edges of a noise-containing spot image. The spot centroid was then extracted according to the ellipse-fitting method, and the method was experimentally verified. As for the data construction of the LDS, to better inhibit the disturbance from random errors and preserve real profile information, a threshold function with high-order differentiability and adaptive wavelet coefficient contractility was constructed based on the hyperbolic tangent function. This function was followed by automatic optimization of related parameters in the denoising process via the particle swarm optimization algorithm, and then this method was verified by simulation. Furthermore, in consideration that the least square fitting method failed to satisfy the requirements for continuity and differentiability of the function at piecewise points, a smoothing algorithm for point cloud data based on the Lagrange multiplier method was raised, and the algorithm was modeled and solved. A real-time calculation of the profile accuracy of the double-headed screw rotor was performed and the machining quality was judged. The on-machine measurement demonstrates that the measuring system developed can complete precise on-machine measurement of the double-headed screw rotor and it has improved the measurement accuracy and efficiency.

## 2. The Point Cloud Data-Processing Algorithm

### 2.1. A New Method for Centroid Extraction of Laser Spots

In terms of the edge extraction through the mathematical morphological method, the images are matched or locally corrected with the predefined structural elements, and this method can inhibit the noise while extracting detailed edge information. However, some detailed features of the image edges will be lost due to the use of single structural elements and their directional and structural unicity in the simultaneous edge extraction and noise-filtering process. Therefore, a novel generalized variable-structural-element morphological edge extraction algorithm is adopted in this section to perform effective edge measurement of the spot images.

f(x,y) is set as a digital image on 2D discrete space *Z*^2^ (integer set), the structural element B(e,l) is a subset on *Z*^2^, namely $B\left(e,l\right)\subset Z^{2}$, and morphological dilation and erosion are respectively defined as follows:

(1) $$(f⊕B)(x,y)=\underset{\left(e,l\right)\in B}{\max}\left[f\left(x-e,y-l\right)+B\left(e,l\right)\right]$$

(2) $$(f⊖B)(x,y)=\underset{\left(e,l\right)\in B}{\min}\left[f\left(x+e,y+l\right)-B\left(e,l\right)\right]$$

The edge image $E_{\phi ,\tau }\left(x,y\right)$ measured through the generalized morphological edge extraction algorithm is:

*E_φ,τ_*(*x*,*y*) = {[*f*(*x*,*y*)⊕*B_φ_*(*x*,*y*)] − [*f*(*x*,*y*)⊖*B_τ_*(*x*,*y*)]} (3)

where $B_{\phi }\left(x,y\right)\left(\phi =1,2,\cdots ,A\right)$ and $B_{\tau }\left(x,y\right)\left(\tau =1,2,\cdots ,C\right)$ are selected as variable structural elements.

The accumulative mean of the edge extraction results was taken as the output based on structural and directional symmetrical and complementary characteristics of the variable structural elements, so as to fully extract detail features in various directions of the image edge and effectively smooth the image noise. The edge image E(x,y) extracted through the generalized variable-structural-element morphological edge extraction algorithm is as below:

(4) $$E\left(x,y\right)=\frac{1}{A}\left\{\sum_{e=1}^{\theta }\left[E_{e,e+\theta }\left(x,y\right)+E_{e+\theta ,e}\left(x,y\right)\right]\right\}$$

where *A* denotes the number of the selected variable structural elements; *θ* denotes the number of the element groups, which are complementary in structure and direction, among the selected variable structural elements. For each determined *e* and *θ*, $E_{e,e+\theta }\left(x,y\right)$ and $E_{e+\theta ,e}\left(x,y\right)$ are edge images calculated for a group of variable structural elements which present structural and directional complementary characteristics through Equation (3).

As indicated in Equations (3) and (4), this algorithm has substituted the simple maximum and minimum solving method for the traditional summation and product operations in a complex template for edge extraction, thus realizing the edge extraction from the image only in one cycle. Moreover, it has strong timeliness with simple calculation and easy parallel processing.

The aforementioned method can be used to rapidly and effectively extract spot edge images. To further improve the positioning accuracy and timeliness of the positioning algorithm, the polynomial interpolating subpixel edge positioning algorithm was used in this section to realize rapid subpixel positioning of the image edge. The spacing in direction *X* and *Y* on a charge-coupled device (CCD) is set as *W* and *V*, respectively, and then three points $E\left(x_{\phi -1},y_{\varsigma }\right)$, $E\left(x_{\phi },y_{\varsigma }\right)$ and $E\left(x_{\phi +1},y_{\varsigma }\right)$ in direction X are taken for the determined edge point $\left(x_{\phi },y_{\varsigma }\right)$ on the edge image E(x,y). Grayscale values of the three points serve as the functions and $x_{\phi -1}$, $x_{\phi }$ and $x_{\phi +1}$ as interpolating base points, which are then substituted into the quadratic polynomial interpolating function ϕ(x). $\frac{d\phi \left(x\right)}{dx}=0$ set, the position *x_s_* of the subpixel with the maximum interpolating function value in direction *X* is solved. Similarly, three points $E\left(x_{\phi },y_{\varsigma -1}\right)$, $E\left(x_{\phi },y_{\varsigma }\right)$ and $E\left(x_{\phi },y_{\varsigma +1}\right)$ are taken in direction *Y* for the same operation. $\frac{d\phi \left(y\right)}{dy}=0$ set, the position *y_s_* of the subpixel in direction *Y* can be solved, so the subpixel position $\left(x_{s},y_{s}\right)$ at the edge point $\left(x_{\phi },y_{\varsigma }\right)$ is solved as:

(5) $$\{\begin{array}{c} x_{s}=x_{\phi }+\frac{E\left(x_{\phi -1},y_{\varsigma }\right)-E\left(x_{\phi +1},y_{\varsigma }\right)}{E\left(x_{\phi -1},y_{\varsigma }\right)-2E\left(x_{\phi },y_{\varsigma }\right)+E\left(x_{\phi +1},y_{\varsigma }\right)}\times \frac{W}{2}\\ y_{s}=y_{\phi }+\frac{E\left(x_{\phi },y_{\varsigma -1}\right)-E\left(x_{\phi },y_{\varsigma +1}\right)}{E\left(x_{\phi },y_{\varsigma -1}\right)-2E\left(x_{\phi },y_{\varsigma }\right)+E\left(x_{\phi },y_{\varsigma +1}\right)}\times \frac{V}{2} \end{array}$$

The subpixel positional information of effective edge points selected on the spot profile based on the generalized variable-structural-element morphological edge extraction algorithm can be obtained through the polynomial interpolating subpixel edge positioning. To locate the spot centroid accurately, ellipse fitting of the edge can be conducted according to the principle of least squares, so as to solve the elliptic center to substitute the spot centroid in the image. The general ellipse equation is as follows:

(6) $$\xi x^{2}+\zeta xy+\psi y^{2}+\delta x+\iota y+\upsilon =0$$

The optimal solutions of parameters ξ,ζ,ψ,δ,ι,υ are solved based on the difference minimum principle, so as to obtain the general ellipse equation. Through the optimal solutions, the coordinates $\left(x_{0},y_{0}\right)$ of the centroid can be calculated as:

(7) $$\{\begin{array}{c} x_{0}=\frac{\zeta \iota -2\psi \delta }{4\xi \psi -\zeta^{2}}\\ y_{0}=\frac{\zeta \delta -2\xi \iota }{4\xi \psi -\zeta^{2}} \end{array}$$

**Figure 1:**
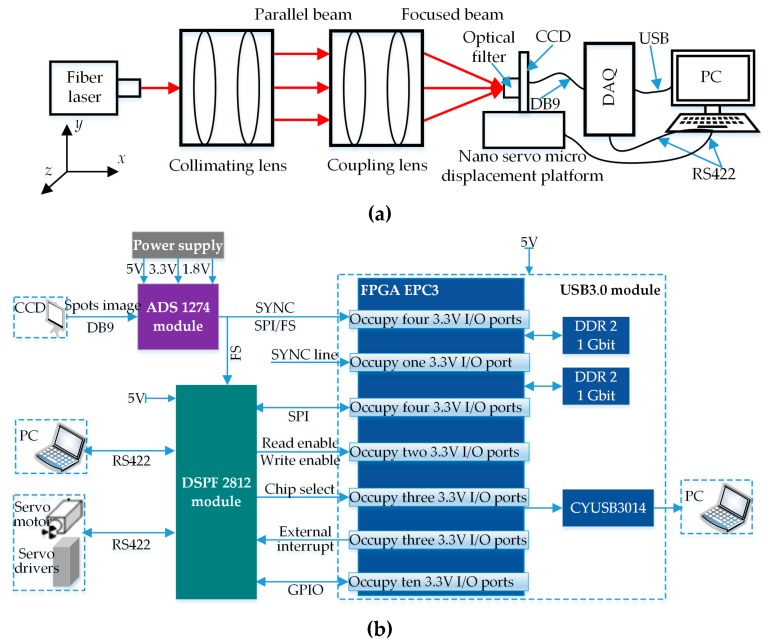
Spot image signal acquisition system. (**a**) Experimental principle schematic diagram. (**b**) Data acquisition (DAQ) board architecture diagram.

This section also designed a spot centroid extraction experiment to verify the effectiveness of the above method. A spot image signal acquisition system was set up on the optical table in this experiment (Figure 1), where Figure 1a is experimental principle schematic diagram and Figure 1b is a data acquisition (DAQ) board architecture diagram. As shown in Figure 1a, this system consists of fiber laser, collimating lens, coupling lens, nano servo micro-displacement platform, linear array CCD, optical filter, DAQ board and PC. The parameters of each component are as follows. A 1550 nm optical fiber sensor is taken as the light source, and luminous power is continuously adjustable. The linear array CCD has a resolution of 4000 pixels, pixel size (spacing) of 7 μm × 7 μm × 7 μm, scanning a frame for 5 ms, sealed in an 80 mm × 80 mm aluminum specimen box. Full width at half maxima of 1500 nm optical fiber is 12 nm. The nano servo micro-displacement platform, with repeated positioning accuracy of 2 nm, can realize precise movement in two directions *x*, *y* and *z*. The PC is configured for Intel Core I7 processor with a 64-bit operating system, 3.8 GHz operating speed and 8 GB memory. In addition, in order to guarantee real-time transmission, saving and processing of experimental signals, a DAQ board was also designed in this section with its architecture as shown in Figure 1b, mainly including a USB3.0 module, ADS1274 module and DSPF2812 module. Furthermore, this DAQ board is connected to PC and nano servo micro-displacement platform via RS422 and controls servo motion of the displacement platform through its DSPF module.

The concrete experimental process is as follows. The fiber laser emitted light beams within the waveband of 1550 nm, which were then turned into approximate parallel lights after passing through collimating and shaping lens assembly, and finally the spot fell on the photosensitive surface of the CCD after focusing through a coupling lens and transmission through an optical fiber. CCD was placed in front of the focal point of the coupling lens so that the radius of the spot falling on the CCD photosensitive surface was regulated with the change of the distance from CCD to lens assembly. As the CCD was placed on the 3D nano micro-displacement platform, the servo motor and servo driver of this platform were controlled through the PC and DAQ board so as to realize nanoscale-precise movement of the CCD and regulate coordinates of the spot falling on the CCD photosensitive surface. The spot image acquired by the CCD was transmitted to the DAQ board via the DB9 interface and processed with the spot centroid extraction method proposed in this section. The result was displayed on the PC with the USB. In the experiment, the output power of the optical laser was regulated to 10 μw and the sampling rate of the DAQ board was set as 1 kHz. The spacing between the lens and CCD and the position of the spot image on the CCD were regulated though the nano micro-displacement servo platform, and as a result, the spot size was adjusted to 30 × 30 pixels and its coordinates as (100, 100).

**Figure 2:**
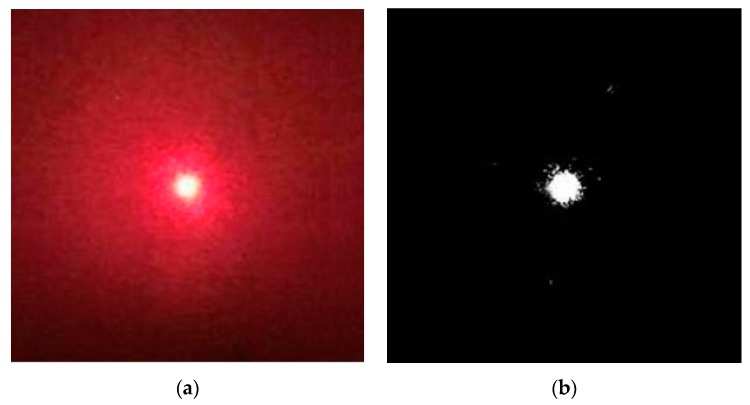
Original spot image acquired through the experiment. (**a**) Real spot image. (**b**) Binarized spot image.

**Figure 3:**
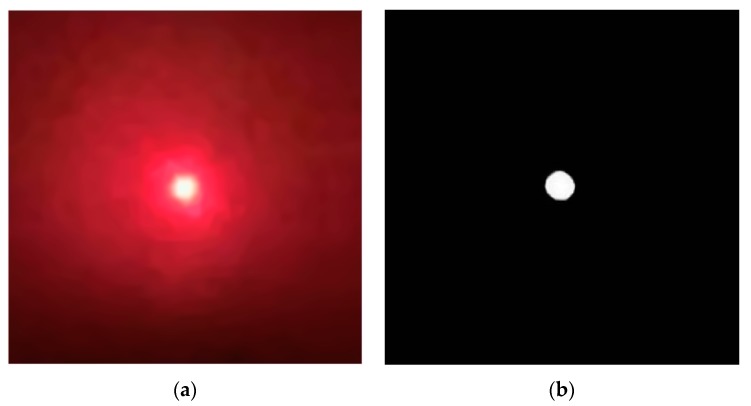
Processed spot image through our method. (**a**) Real spot image. (**b**) Binarized spot image.

The spot image acquired in the experiment is shown in Figure 2, where Figure 2a is a real image and Figure 2b is a binarized image. As shown in Figure 2, speckle noises, certain random noises and background radiation noises exist in the spot image acquired in practical engineering, and its binarized image is processed as follows: initially, the effective edge of the spot image is measured through the generalized variable-structural-element morphological edge extraction algorithm. The measured effective edge points are then positioned according to the polynomial interpolating subpixel edge-positioning algorithm. Ellipse fitting of all positioning points is finally conducted via the least square method, so as to solve the centroid of the ellipse, namely the spot centroid. The spot image processed as per the above method is shown in Figure 3, where Figure 3a is a real image and Figure 3b is its binarized image. As shown in Figure 3, the spot image processed through the method in this section is more regular and smoother. This method has not only fully extracted detailed edge information of the spot image but also effectively inhibited noises in the image, thus being able to realize the subpixel centroid extraction of the noise-containing spot.

In addition, in order to further highlight advantages of the proposed method in the aspect of extraction accuracy of the spot centroid, the grayscale centroid method, Gaussian fitting method, ellipse fitting method and the method in this section are used to extract the original spot centroid in Figure 2, and then centroid extraction accuracies of the four methods are compared as shown in Table 1. In Table 1, root-mean-square value (RMS) and peak valley (PV) of the centroid extraction deviation are used to analyze quantitatively the centroid extraction accuracies (in pixels) achieved by the four methods, where PV represents maximum deviation and RMS denotes fluctuation of the deviation. All data in the table are mean values of 10-times experimental results. Based on the comparison in Table 1, the centroid extraction via the Gaussian fitting method has the highest RMS and PV with the poorest centroid accuracy, influenced by the noises in the original spot image. The centroid extraction via the grayscale centroid method has the second largest RMS and PV with relatively poor centroid accuracy influenced by the complexity and irregularity of the original spot image. The centroid extraction through the ellipse-fitting method has the third largest RMS and PV with ordinary centroid accuracy influenced by the intensity asymmetry and edge blur factors of the original spot image. However, the proposed method has successfully solved the large ellipse-fitting error under intensity asymmetry and edge blur with both RMS and PV reaching the minimum values, so its centroid extraction accuracy is the highest.

### 2.2. A New Wavelet Threshold-Denoised Method of Point Cloud Data

In the laser scanning measurement of the double-headed screw rotor profile, denoising is necessary as noise defects unavoidably exist in the LDS-acquired point cloud data under the influence of local morphology of the spiral curved surface as well as the defects of the measuring system itself and measuring environment. The wavelet threshold denoising algorithm was used in this section to process the above data. Besides the traditional hard threshold function and soft threshold function, there is also the improved threshold function proposed in reference [29]. The three threshold functions are as follows:

The hard threshold function:

(8) $$\eta \left(x,\lambda \right)=\{\begin{array}{c} x,\left|x\right|\geq \lambda \\ 0,\left|x\right|\leq \lambda \end{array}$$

The soft threshold function:

(9) $$\eta \left(x,\lambda \right)=\{\begin{array}{l} x-\lambda \;,x\geq \lambda \\ 0\;,\left|x\right|<\lambda \\ x+\lambda ,x\leq -\lambda \end{array}$$

The threshold function improved by reference [29]:

(10) $$\eta \left(x,\lambda \right)=\{\begin{array}{l} x-0.5\frac{\lambda^{s}k}{x^{s-1}}+\left(k-1\right)\lambda \;,x>\lambda \\ 0.5\frac{k{\left|x\right|}^{s+2-k}}{\lambda^{s+2\left(1-k\right)/k}}\cdot \mathrm{sgn}\left(x\right)\;,\left|x\right|\leq \lambda \\ x+0.5\frac{k{\left(-\lambda \right)}^{s}}{x^{s-1}}-\left(k-1\right)\lambda ,x<-\lambda \end{array}$$

where *x* denotes the wavelet decomposition coefficient; *λ* denotes the wavelet threshold: $\lambda =\sigma \sqrt{2\log\left(N\right)}$; *σ* denotes the standard deviation of the noise; *N* denotes the signal length. Under normal circumstances, the standard deviation of a noise signal is uncertain, so it should be estimated according to $\sigma =\frac{\mathrm{MEDIAN}\left|d_{1,k}\right|}{0.6745}$. $\mathrm{MEDIAN}\left|d_{1,k}\right|$ represents the mean of high-frequency wavelet coefficient at the first layer. In addition, *s* and *k* in Equation (10) are adjustment factors of the threshold function; *s* decides the shape of the threshold function while *k* determines the asymptotic line of the threshold function, and its value ranges from 0 to 1.

The traditional wavelet threshold method still has some prominent defects, even though it has a certain effect on data denoising: only a large wavelet coefficient is preserved in the denoising process through the hard threshold function while the small wavelet coefficient is given up, which leads to interruption at x=λ. As a result, the reconstructed data may fluctuate and affect the denoising effect. In the denoising through the soft threshold function, large wavelet coefficients are compressed with favorable consistency and remarkable denoising effect. However, a constant deviation exists between the processed wavelet coefficient and original wavelet coefficient due to discontinuity of its derivatives, affecting the approximation degree of the reconstructed data to original data, thus giving rise to distortion of the edge data. Besides, both hard and soft threshold functions have abandoned wavelet coefficients smaller than their thresholds in the denoising process and, consequently, useful data blended in the noises are filtered, generating a certain deviation between the reconstructed data and the original data. The improved threshold function in reference [29] has satisfied the continuity requirement but still preserved the denoising deviation.

For the aforementioned defects of the traditional wavelet threshold function, a new-type adaptive wavelet threshold function was constructed in this study. This function has not only preserved the advantages of the traditional threshold function but also guaranteed the continuity of the function itself and its derivatives and, meanwhile, it has avoided data distortion. The new wavelet threshold function is:

(11) $$\eta \left(x,\lambda \right)=\{\begin{array}{l} x-0.48\frac{{\left(0.1+\tanh\left(x+1\right)\lambda \right)}^{a}}{x^{a-1}}+\tanh\left(x+1\right)\left(u-1\right)\lambda ,\left|x\right|>\lambda \\ 0.48\frac{{\left|x\right|}^{b}}{\lambda^{b-1}}\tanh\left(x+1\right)\;,\left|x\right|\leq \lambda \end{array}$$

where tanh is a hyperbolic tangent function, parameters a, b and u are regulatory factors, and the denoising sensitivity of the threshold function in practical engineering can be enhanced by adjusting the parameter values. *a* and *b* (*a* > 0, *b* > 0) are two shape control parameters of the new-type threshold function; *u* (0 < *u* < 1) is a control parameter for approximation degree of the new-type threshold function. If *u* = 1, the threshold function is more approximate to the hard threshold function, and if *u* = 0, the threshold function is more approximate to the soft threshold function.

From the above function form and parameter change, the constructed new-type threshold function has high-order differentiability and adaptive wavelet coefficient contractibility. While preserving denoising advantages of hard and soft threshold functions, it can also always maintain the continuity of the threshold function at *λ* by regulating the parameter *u*, so as to prevent oscillation and avoid the deviation generated in the wavelet coefficient decomposition. Therefore, it can be forecasted that this new-type threshold function can not only enhance the capability of wavelet denoising but also improve the elimination phenomenon of real data mixed in random noises.

Through the above analysis results, the values of regulatory factors *a*, *b* and *u* are of vital importance to the denoising effect of the constructed new-type threshold function. Hence, an automatic optimization method for regulatory factors based on the particle swarm algorithm is also raised in this section, and the concrete steps are as follows:

Step 1: Initializing the search space and position. The three-dimensional vector consisting of *a*, *b* and *u* was taken as the input of particle position. The search space of *a* and *b* was initialized as [1,10] with step size of 1. The search space of *u* was initialized as (0,1) with step size of 0.1. Under normal circumstances, the size of the particle swarm is twice or more than twice as many as the number of searched dimensions, and it was taken as 10 in this section.

Step 2: Calculating the particle fitness *Fit*[*i*] according to the fitness function.

Signal-to-noise ratio (SNR) and mean squared error (MSE) are the main indicators used to measure the denoising performance as shown in Equations (12) and (13), respectively.

(12) $$\mathrm{SNR}=10\mathrm{lg}\frac{\sum_{i=1}^{N}x{\left(i\right)}^{2}}{\sum_{i=1}^{N}\left(x\left(i\right)-\hat{x}{\left(i\right)}^{2}\right)}$$

(13) $$\mathrm{MSE}=\frac{1}{N}{\sum_{i=1}^{N}\left(x\left(i\right)-\hat{x}\left(i\right)\right)}^{2}$$

where x(i) is original data; $\hat{x}\left(i\right)$ is post-denoising data.

As the particle swarm algorithm determines a global minimum value, MSE is taken as the fitness function in this section.

Step 3: Updating the position and speed of each particle according to Equations (14) and (15).

(14) $$v_{id}=w^{*}v_{id}+c_{1}r_{1}\left(p_{id}-x_{id}\right)+c_{2}r_{2}\left(p_{gd}-x_{id}\right)$$

(15) $$x_{id}=x_{id}+v_{id}$$

where the defining and initializing methods for constants and variables are as follows:

(1) Inertia weight *w*: the linearly decreasing weight method means that the inertia weight decreases progressively in a linear way, and the change mode is:
  (16) $$w=w_{\max}-\frac{t^{*}\left(w_{\max}-w_{\min}\right)}{T}$$
  where *w*_max_ is maximum inertia weight; *w*_min_ is minimum inertia weight; *t* is the current number of iteration times; *T* is total number of iteration times. Here $w_{\max}=0.8$, $w_{\min}=0.3$ and *T* = 100. The learning factors *c*_1_ and *c*_2_ are two fixed constants with value range being [0,4], and *c*_1_ = *c*_2_ = 1.5 is taken in this section. *r*_1_ and *r*_2_ are two random constants within [0,1].
(2) *x_id_* and *v_id_* represent the *d*-dimensional position and speed of the particle *i*, respectively.
(3) The optimal position searched for the particle *i* so far is called individual extremum and recorded as $p_{\mathrm{best}}=\left(p_{i1},p_{i2},\cdots ,p_{id}\right),i=1,2,\cdots ,10$. The optimal position searched for the whole particle swarm so far is a global extremum recorded as $g_{\mathrm{best}}=\left(p_{g1},p_{g2},\cdots ,p_{gd}\right)$.

Step 4: Restricting the updated position and speed. If the new speed of a particle goes beyond the range of [*v*_max_,*v*_min_], it is set as a new boundary. The new position can be processed in the same way.

Step 5: Comparing the fitness value *Fit*[*i*] and individual extremum *p*_best_(*i*) for each particle. If *Fit*[*i*] > *p*_best_(*i*), *Fit*[*i*] is used to replace *p*_best_(*i*). *Fit*[*i*] and *g*_best_(*i*) are compared in the same way, and if *Fit*[*i*] > *g*_best_(*i*), *Fit*[*i*] is used to replace *g*_best_(*i*).

Step 6: Exiting if the end condition or maximum number of iterations is reached, otherwise returning to Step 2.

**Figure 4:**
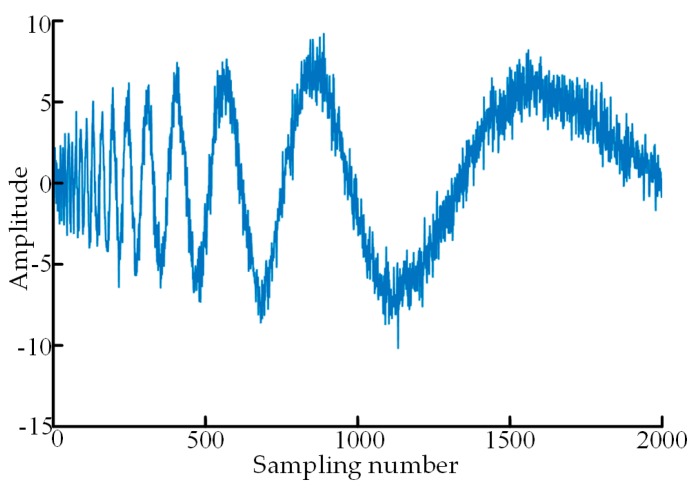
The noisy point cloud data in simulation verification.

**Figure 5:**
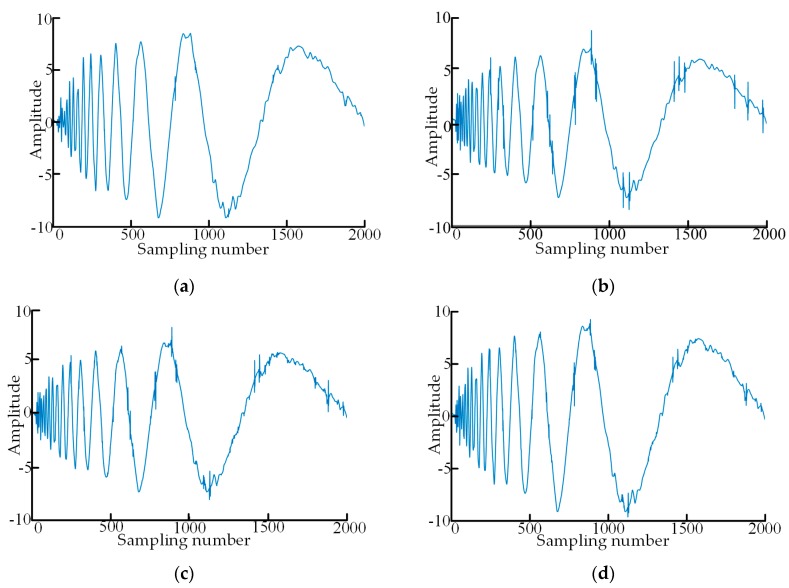
The comparison of noise reduction results in simulation. (**a**) Data after noise reduction by soft threshold function; (**b**) data after noise reduction by hard threshold function; (**c**) data after noise reduction in reference [29] function; (**d**) data after noise reduction by new threshold function.

In order to verify the denoising performance of the proposed new-type adaptive threshold function, a simulation verification was designed in this section. A group of point cloud data superposed with Gaussian white noises were used in the simulation, where the number of sampling points is 2000, the superposed Gaussian white noises follow N (0,1) distribution, and SNR of the noise-containing point cloud data is 4 as shown in Figure 4. The point cloud data in Figure 4 are denoised with the traditional soft and hard threshold functions, the threshold function improved in reference [29], and the adaptive threshold function constructed in this paper, respectively. Sym4, sym6 and sym8 in the Symlets wavelet system and db6, db8 and db10 in the Daubechies wavelet system were selected as wavelet-generating functions to denoise the above noise-containing data, and after a comparison of the denoising results, sym6 wavelet was used to perform four-layer decomposition of the data. The above particle swarm algorithm was used to automatically determine the optimal regulatory factors according to the heuristic threshold rules (Heursure rules). The denoising results of the four groups of wavelet threshold functions are shown in Figure 5.

To compare the denoising effects of traditional soft and hard threshold functions and the aforementioned two threshold functions accurately, unified objective evaluation criteria should be introduced. Two evaluation indicators SNR and MSE for the denoising performance were used for a quantitative analysis of the denoising effects. The definitions are as follows: the higher the SNR of point cloud data with denoising completed and the smaller the MSE, the more approximate the post-denoising data is to the original data and, meanwhile, the denoising effect is better. The unit of SNR is dB. The concrete values of evaluation criteria for the four threshold functions after denoising are listed in Table 2.

Through comparison of the four graphs in Figure 5, the soft threshold function has strong denoising capability with smooth denoising data, but it eliminates the real detailed information in the data seriously. The hard threshold function has poor denoising capability with the waveform fluctuating to some extent and, moreover, it cannot filter random errors very well with insignificant denoising effect and obvious noises. The improved threshold function in reference [29] solves the oscillation phenomenon caused by the discontinuity of the hard threshold function at x=λ, improves the data denoising level and elevates the performance indicators. However, the denoising ability is enhanced by sacrificing some real detailed data information in its implementation process. The proposed new-type adaptive wavelet threshold function has successfully solved the above problems. It has not only avoided the discontinuous oscillation phenomenon but also controlled the mistaken deletion of high-frequency signals in real information well and improved the data reconstruction accuracy. It has achieved remarkable effects in preserving signal authenticity and integrity as well as good denoising effect. Furthermore, as shown in Table 2, the denoising effect for the data by the proposed new-type adaptive wavelet threshold function is superior to those by other threshold functions.

### 2.3. A Fairing Processing Method of Point Cloud Data

**Figure 6:**
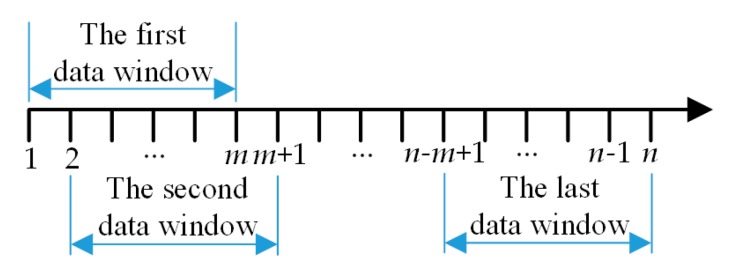
The schematic segment curve fitting.

As the size of the LDS-scanned profile data of the double-headed screw rotor is large, it is not appropriate to perform the global fitting operation, and it is necessary to conduct piecewise fitting at different data points for multiple times. However, the above method failed to satisfy the continuity and differentiability requirements for the function at piecewise points, so a smoothing method for point cloud data based on the Lagrange multiplier method was put forward in this section. The piecewise curve fitting was implemented in windows in this section. A polynomial fitting equation could be obtained by fitting the data in each window just for a single time, the fitting curve between the first two data was then output, the window was then moved backward for one data point, and the next window was constituted for the next fitting until the last window is fitted as shown in Figure 6. Among them, *m* data are used to fit *n* data points for a window, with a total of *n* − *m* + 1 windows.

The interval of point cloud data is set as [*x*_1_, *x_n_*], *n* data points are $\left(x_{j},y_{j}\right),j=1,2,\cdots ,n$, and then the approximating polynomial of the windows is:

(17) $$g\left(x\right)=\{\begin{array}{c} g_{1}\left(x\right),x_{1}<x<x_{m}\\ g_{2}\left(x\right),x_{2}<x<x_{m+1}\\ \vdots \;\vdots \;\\ g_{n-m+1}\left(x\right),x_{n-m+1}<x<x_{n} \end{array}$$

where the least squares fitting in traditional sense was adopted in the windows to obtain the piecewise fitting polynomial.

The polynomial at the window q(q=1,2,⋯,n−m+1) is:

(18) $$g_{q}\left(x\right)=\sum_{h=0}^{M}a_{qh}x^{h},x_{q}<x<x_{q+m-1}$$

where $a_{q0},a_{q1},\cdots ,a_{qM}$ are undetermined coefficients.

To ensure that at *x_q_*, the curve is continuous when *g_q_*_-1_(*x*) is transited to *g_q_*(*x*), the functional values of the two pieces of the curve are made equal at *x_q_*, namely adding the endpoint constraint condition *g_q_*_-1_(*x_q_*) = *g_q_*(*x_q_*). In the meantime, to ensure that at *x_q_*, the curve is smooth when *g_q_*_-1_(*x*) is transited to *g_q_*(*x*), the first-order derivatives of the two pieces of curve are also made equal at *x_q_*, namely adding the endpoint constraint condition *g_q_*_-1′_(*x_q_*) = *g_q_*’(*x_q_*).

The above mathematical model is a constraint problem with extremums. The Lagrange multiplier method was adopted in this section to transform it into an unconditional extremum problem for solving. The window *q* taken for example, the Lagrange multipliers *μ*_1_ and *μ*_2_ were introduced, $H_{q}=g_{q}\left(x_{q}\right)-g_{q-1}\left(x_{q}\right)$ and $H_{q}{}^{\prime }=g_{q}{}^{\prime }\left(x_{q}\right)-g_{q-1}{}^{\prime }\left(x_{q}\right)$ were set, and then the boundary constraint condition was $H_{q}=0,H_{q}{}^{\prime }=0$, so the Lagrange equation of this mathematical model was obtained as below:

(19) $$L=\sum_{j=q}^{q+m-1}{\left(\sum_{h=0}^{M}a_{qh}x_{j}{}^{h}-y_{j}\right)}^{2}+2\mu_{1}H_{q}+2\mu_{2}H_{q}{}^{\prime },\left(x_{q}<x_{j}<x_{q+m-1}\right)$$

The fitted curve can be approximate to the measured value as far as possible if the Lagrange functional value *L* is minimized by changing the coefficient *a_qh_*. Therefore, *L* is made to solve the partial derivative of each coefficient and its partial derivative is set as zero, namely:

(20) $$\frac{\partial L}{\partial a_{qh}}=2\sum_{j=q}^{q+m-1}\left(\sum_{h=0}^{M}a_{qh}x_{j}{}^{h}-y_{j}\right)x_{j}{}^{f}+2\mu_{1}\varphi_{f}\left(x_{q}\right)+2\mu_{2}\varphi_{f}{}^{\prime }\left(x_{q}\right)$$

where $\varphi_{f}\left(x\right)=x^{f},f=1,2,\cdots ,M$. Equation (20) is set as zero and written into the following matrix form:

(21) $$\left[\begin{array}{cc} S & S_{1}\\ S_{2} & 0 \end{array}\right]\left[\begin{array}{c} A\\ \mu \end{array}\right]=\left[\begin{array}{c} G\\ G_{1} \end{array}\right]$$

where ***S*** = $\left[\begin{array}{llll} \langle \varphi_{0},\varphi_{0}\rangle & \langle \varphi_{0},\varphi_{1}\rangle & \cdots & \langle \varphi_{0},\varphi_{M}\rangle \\ \langle \varphi_{1},\varphi_{0}\rangle & \langle \varphi_{1},\varphi_{1}\rangle & \cdots & \langle \varphi_{1},\varphi_{M}\rangle \\ \vdots & \vdots & & \vdots \\ \langle \varphi_{M},\varphi_{0}\rangle & \langle \varphi_{M},\varphi_{1}\rangle & & \langle \varphi_{M},\varphi_{M}\rangle \end{array}\right]$, and $\langle ,\rangle$ is their internal product; ***S*_1_** = $\left[\begin{array}{ll} \varphi_{0}\left(x_{q}\right) & \varphi_{0}{}^{\prime }\left(x_{q}\right)\\ \varphi_{1}\left(x_{q}\right) & \varphi_{1}{}^{\prime }\left(x_{q}\right)\\ \vdots & \vdots \\ \varphi_{M}\left(x_{q}\right) & \varphi_{M}{}^{\prime }\left(x_{q}\right) \end{array}\right]$; ***S*_2_** = ***S*_1_**^T^; ***A*** = ${\left[a_{q0},a_{q1},\cdots ,a_{qM}\right]}^{T}$; ***μ*** =${\left[\mu_{1},\mu_{2}\right]}^{T}$; ***G*_1_** = ${\left[g_{q-1}\left(x_{q}\right),g_{q-1}{}^{\prime }\left(x_{q}\right)\right]}^{T}$; ***G*** = ${\left[\left(F,\varphi_{0}\right),\left(F,\varphi_{1}\right),\cdots ,\left(F,\varphi_{M}\right)\right]}^{T}$, ***F*** = ${\left[y_{q},y_{q+1},\cdots ,y_{q+m-1}\right]}^{T}$ and ***φ****_f_* = ${\left[x_{q}{}^{f},x_{q+1}{}^{f},\cdots ,x_{q+m-1}{}^{f}\right]}^{T},f=0,1,\cdots ,M$.

**Figure 7:**
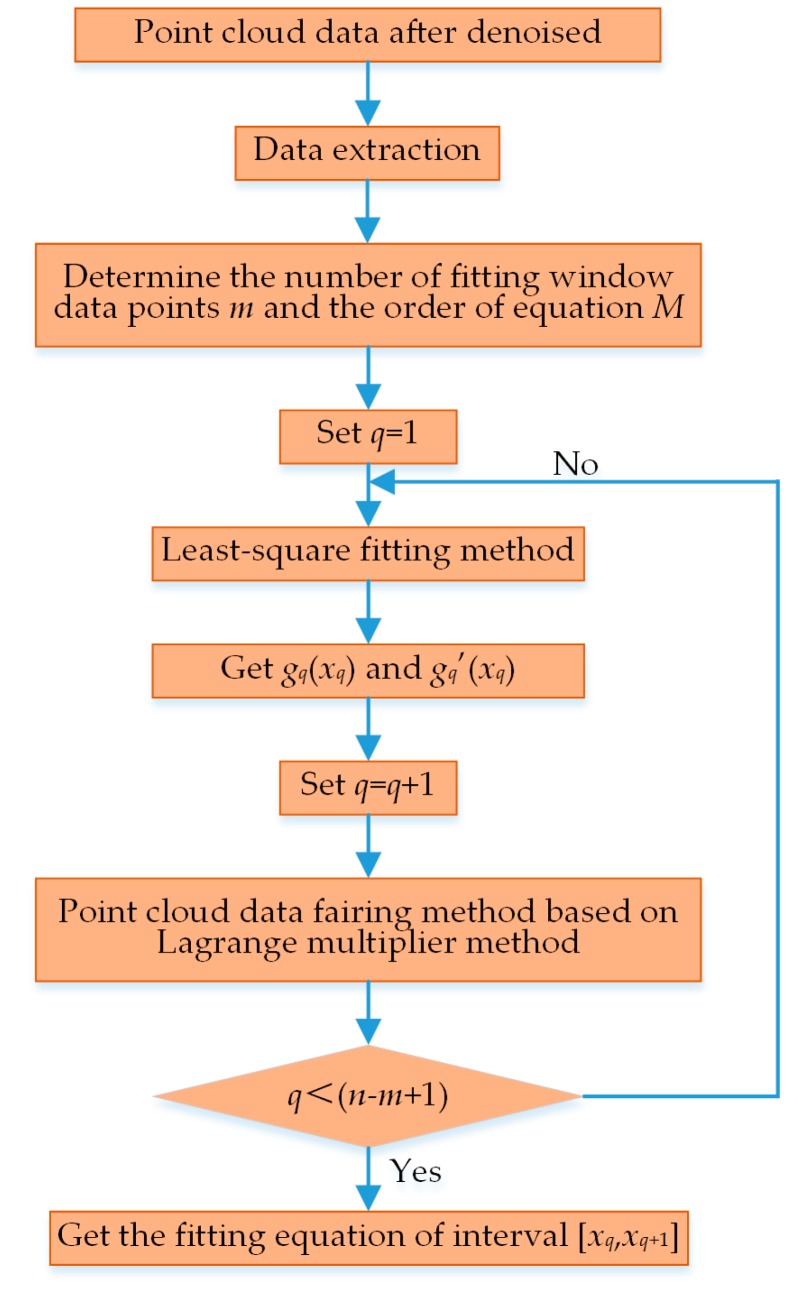
The flowchart of fairing processing for point cloud data.

After the matrix Equation (21) is solved, the coefficient in the Lagrange function $a_{qh}\left(h=1,2,\cdots ,M\right)$ and *μ*_1_ and *μ*_2_ can be solved so as to obtain the fitted curve between data points $\left[x_{q},x_{q+1}\right]$. The windows are solved successively according to this method to obtain the global fitted curvilinear equation. The flowchart of this algorithm is shown in Figure 7.

## 3. The Measuring System Configuration

**Figure 8:**
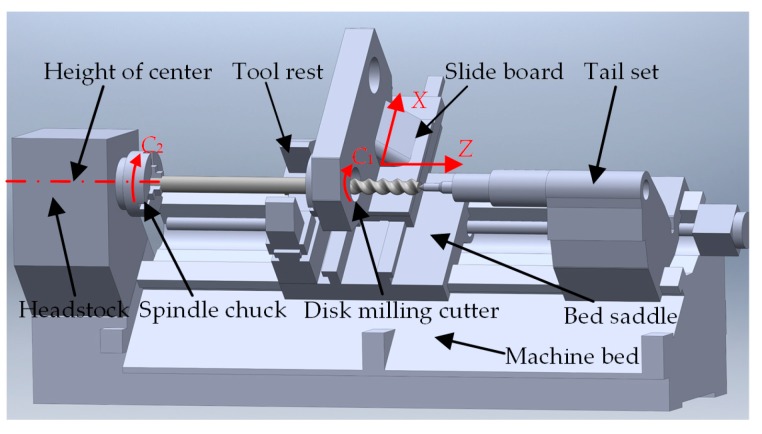
Whirlwind milling machine with LXK100 four-axis double-headed screw rotor.

The whirlwind milling machine structure with the LXK100 double-headed screw rotor is shown in Figure 8. According to the formation mechanism of a spiral curved surface, this special-purpose machine needs four motion axes: rotation axis *C*_2_ of workpiece around its own axis, rotation axis *C*_1_ of tool nose around the cutterhead axis, feed axis *X*, which controls the distance from spin axis of the cutterhead to spin axis of the workpiece, and feed axis *Z*, which controls the axial motion of the workpiece. In the machining process, axes *C*_1_, *C*_2_, *X* and *Z* should be interlinked, and all arrows in the figure point at the forward direction, where *Z*-directional positioning accuracy of the machine is 0.01 mm/500 mm and the straightness of the guide rail is 0.05 mm/1000 mm. The basic structure of the whirlwind milling machine with a LXK100 double-headed screw rotor is summarized as follows: horizontal-type oblique tool bed structure is used, at the left of the tool bed is a spindle box, two guide rails are arranged from the spindle box to tail table along the direction of axis *Z*, sliding bases are placed on the guide rails, and the motion direction of the sliding plate on each sliding base is axis *X*, which is perpendicular to axis *Z*. One end of the workpiece is clamped in the chuck of the principal axis while the other end is fixed by the top of the tail table. The facing cutter is installed on the sliding plate. In the direction perpendicular to the installation surface of the sliding plate, the workpiece axis has the same height as the rotation axis of the cutterhead, and a follow rest is placed at the left side of the facing cutter to enhance the cutting stability.

**Figure 9:**
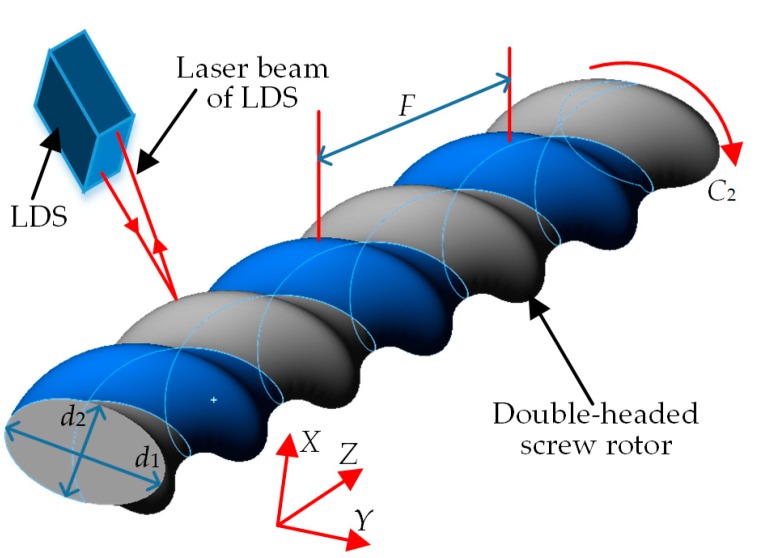
Laser-displacement sensor (LDS)-based on-machine measuring scheme of double-headed screw rotor.

In the LDS-based on-machine measuring scheme for the double-headed screw rotor (Figure 9), the spiral curved surface of the double-headed screw rotor is formed through the motion of the rotor generatrix (cross-sectional profile of the ellipse) along the spiral line, and its major axis *d*_1_, minor axis *d*_2_ and screw lead *F* are the primary profile parameters. Among them, the screw lead *F* is the length for one head on the generatrix to move for one cycle along the spiral line, and as a general rule, the screw pitch *w* = *F*/2 is used as its substitutive test parameter. The LDS can be installed on the side wall of the follow rest by regulating the mechanical bracket, and its laser beam is parallel to the motion direction of axis *X* of the machine and passes through the workpiece axis. The LDS is then moved to the measured position through the motion of axes *X* and Z. In the measuring process of major axis *d*_1_ and minor axis *d*_2_, the LDS remains unmoved while the workpiece rotates along the axis *C*_2_ for one cycle, so as to obtain the cross-sectional profile data. In the measuring process of the screw pitch *w* of the workpiece, the workpiece is unmoved while the LDS moves for a corresponding length along axis *Z*, so as to obtain the axial profile data of the rotor. The on-machine measuring process is consistent with the manual measurement mode, namely it is necessary to measure the major axis and minor axis of only one cross section within a screw pitch.

**Figure 10:**
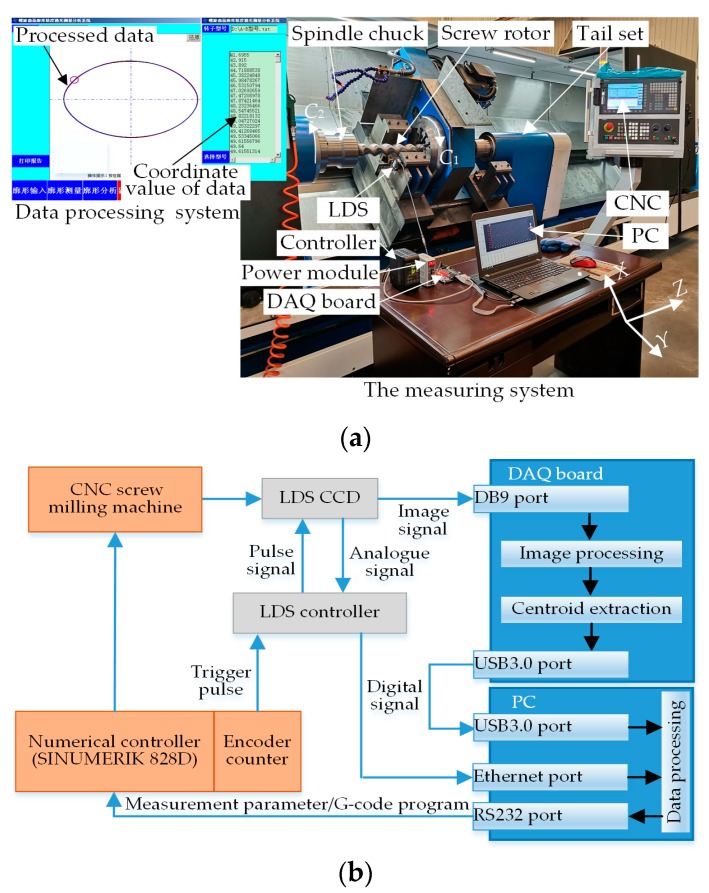
LDS-based on-machine measuring system of double-headed screw rotor. (**a**) Set-up diagram of on-machine measuring system; (**b**) signal flowchart of on-machine measuring system.

The LDS-based on-machine measuring system of the double-headed screw rotor is shown in Figure 10, where Figure 10a is the set-up diagram of the on-machine measuring system and Figure 10b is the signal flowchart of the on-machine measuring system. In Figure 10a, the whole on-machine measuring system consists of three parts: measurement, data processing and computer numerical control (CNC), where the measuring part includes the LDS and its controller. The LDS model is KEYENCE LKH080, its measuring range, resolution, repeatability, linearity and sampling frequency are ±18 mm, 0.1 μm, ±0.02% F.S. and 1 KHz, respectively, and its communication mode includes RS232 and Ethernet. As the core of the whole system, the data-processing part includes the functions of measuring parameters setting, measured data processing, measuring results determination and compensation calculation. The data processing part consists of a PC and DAQ board, of which the parameters are identical with those in Section 2.1, so they will not be described in detail here. SIEMENS SINUMERIK 828D CNC is the execution part of the whole closed measurement-analysis-compensation process. The measuring process of this system is as follows: Before the measurement, the measuring parameters needed are set via the data-processing part, e.g., encoder trigger frequencies of axes *C*_2_ and *Z*, rotation speed of principal axis, feed speed of axis *Z* and model of the measured screw, and the aforementioned parameters are synchronized to the CNC system via the RS232 serial port. In the measuring process of major axis *d*_1_, minor axis *d*_2_ and screw pitch *w*, the rotary encoder of axis *C*_2_ and lead screw encoder of axis *Z* are connected to the external signal output end of the sensor according to the above measuring scheme. When the CNC is driven, the encoders will transmit a pulse signal with a certain frequency to this input end via the controller, and the LDS will send the signal under effective edge triggering of the pulse. In order to compare the improvement effect of the spot centroid extraction method in Section 2.1 on the LDS data acquisition accuracy, the LDS output signal here is divided into two types: one type is the spot image signal acquired by CCD, which is transmitted to DAQ via a DB9 interface. The acquired spot image is processed through the generalized variable-structural-element morphological edge extraction method, rapid polynomial interpolating subpixel edge-positioning algorithm and ellipse fitting method, and the spot centroid is then extracted. Subsequently, the obtained centroid position is transmitted to the PC in the form of digital signal via USB3.0 interface. In the PC, the two groups of acquired signals are denoised and fitted with the methods in Section 2.2 and Section 2.3, respectively. Next, the required parameters are calculated according to the fitted profile, with the machining quality judged, and the result is transmitted to the CNC system via RS232 serial port. Afterwards, cutter compensation and turning correction are performed for the disqualified workpiece, and the compensation is saved into CNC public variables so that it can be invoked at any time in the cutting correction process in order to realize the automatic compensation function. Thus far, the whole closed-loop measurement process is completed and the signal flowchart of the on-machine measuring system is shown in Figure 10b.

**Figure 11:**
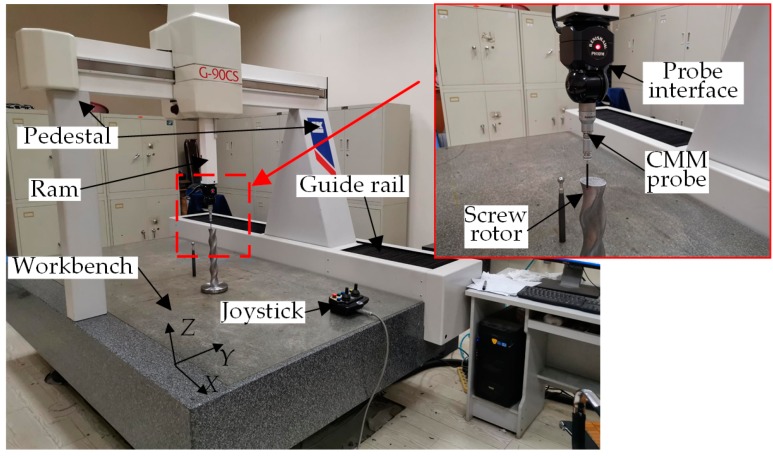
Coordinate measuring machine (CMM) measurement experiment of double-head screw rotor.

In order to verify the capability of the on-machine measuring system, LKG90CS CMM is used to comparatively measure the same cross section of the double-headed screw rotor. The CMM system includes the CNC system, measuring system and PC (Figure 11). The PC is installed with measuring software dedicated for spiral curved surfaces. In the experimental measuring process, the screw part intercepted from the whole screw rotor is installed on the CMM workbench, followed by automatic calibration of the probe through a special software according to the diameter and contact direction of the selected pinpoint. To complete the measurement, it is firstly necessary to import the CAD model of the to-be-measured screw rotor into the software. The closed-line scanning method is then used to determine the starting point and direction of scanning on the CAD model. Scanning control mode and scanning step size are selected in the parameter setting. Finally, the measuring program command is transmitted to the CNC system. The CMM measuring system starts automatic measurement. After the measurement is completed, the computer calculates the corresponding measured data based on the measuring results transmitted by the CNC system. As the measurement accuracy of the CMM is higher than those of other measuring methods, the measured data are taken as real data of actually machined screws.

## 4. Results and Discussion

**Figure 12:**
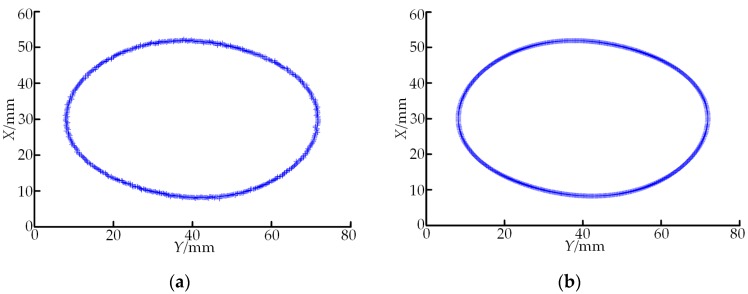
Profile data of double-headed screw rotor. (**a**) Original profile data acquired through the method in Section 2.1; (**b**) profile data denoised through the method in Section 2.2.

**Figure 13:**
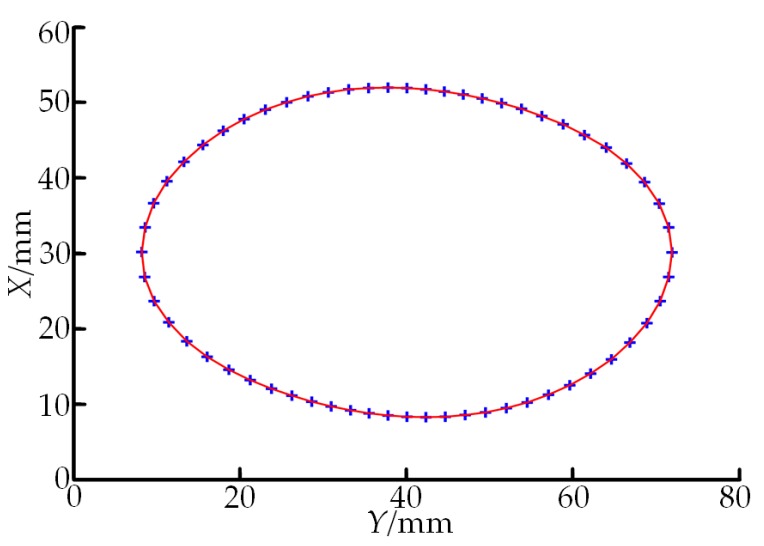
Profile of double-headed screw rotor after smoothing and fitting through the method in Section 2.3.

The parameters of the spiral curved surface, which was once milled on the whirlwind milling machine with the LXK100 four-axis double-headed screw rotor, were measured according to the designed on-machine measuring system. In the cross-sectional profile data of the double-headed screw rotor scanned according to the method in Section 2.1 (Figure 12a), a total of 600 discrete points were acquired. It can be seen that influenced by factors such as local morphology of the spiral curved surface, defects of the measuring system itself and measuring environment, certain random noises are contained in the LDS-contained data. The measured point cloud data are denoised through the method in Section 2.2, and the post-denoising cross-sectional profile of the double-headed screw rotor is shown in Figure 12b. Through a comparison between Figure 12a,b, the proposed wavelet threshold denoising method has excellent detailed information preserving capability while maintaining a high denoising capability, and the post-denoising profile data are obviously smooth. The post-denoising data are fitted through the point cloud data smoothing method based on the Lagrange multiplier method in Section 2.3. In order to improve the efficiency, the aforementioned 600 points are simplified and reduced before the data fitting, namely one among every 10 points is taken as the fitting data. In addition, the number of fitted window points and the equation order should be determined. The number of wind points is generally selected as four to eight pairs of data point coordinates. If the number of data points fitted for a single time is too large, the fitting effect is slightly poorer, but if it is too small, the overall smoothness of the curve cannot reach the optimal effect and, moreover, the calculated quantity for data processing is enlarged. In this experiment, five points are selected as the one-time fitting window. The order of a polynomial is usually selected as two to four orders. Through repeated tests, the expected processing effect is reached when the second-order polynomial is a fitting function. The fitting result is shown in Figure 13, where “+” represents the discrete data after denoising and reduction and the red solid line denotes the fitted curve. As shown in Figure 13, scanning data fits in well with the fitted profile and all pieces of the fitted curve have good recursive properties. Hence, this algorithm can contribute to the continuity of the curve and global continuity of the first-order derivative while preserving the original properties of the curve, manifesting that the processed curve is smooth with good effect. Meanwhile, all kinds of defects brought by high-order curve fitting are avoided and the expected smoothing effect on point cloud data is realized. In the end, the profile parameters of the double-headed screw rotor, major axis *d*_1_ and minor axis *d*_2_, are calculated according to the fitted curve, followed by the judgment of the machining quality based on the parameter calculation results. For a disqualified workpiece, its compensation is given for automatic milling correction. The processing method for measured screw pitch *w* is identical with that for major axis *d*_1_ and minor axis *d*_2_, so it will not be described in detail.

**Figure 14:**
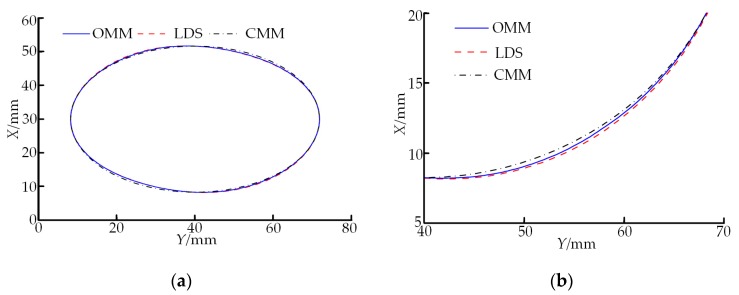
Comparison of measuring results of three methods. (**a**) Overall graph of measuring results; (**b**) locally amplified graph of measuring results.

To verify the use effect of the proposed on-machine measuring method (OMM) in the measuring process of the spiral curved surface, the direct LDS-based measuring method and CMM-based measuring method were used to measure comparatively the same double-headed screw rotor along the same path in Section 3. The measuring results are shown in Figure 14, where Figure 14a is the comparison chart of the overall measuring results and Figure 14b is the locally amplified comparison chart of the measuring results. In comparison with the direct LDS-based measuring method, the profile measured through the OMM method is more approximate to the measuring result of CMM. This outcome indicates that the LDS data acquisition accuracy can be improved in accordance with the proposed method in Section 2.1, that is, the generalized variable-structural-element morphological edge extraction algorithm is first used to realize effective edge measurement of the spot image and then the polynomial interpolating subpixel edge positioning algorithm is used to realize rapid subpixel positioning. Ultimately, the centroid extraction is conducted through the ellipse-fitting algorithm.

To further verify the OMM measurement accuracy, mean values of various parameters measured 10 times are compared with the results obtained through other measuring methods, and the comparison results are shown in Table 3. For LDS 1, the data directly measured by the LDS are directly fitted through the method in Section 2.3 without denoising through the method in Section 2.2. For LDS 2, the data directly measured by the LDS are denoised through the method in Section 2.2 and then fitted through the method in Section 2.3, and error is the difference value between OMM result and CMM result. Through the comparison data of screw parameters in Table 3, all difference values are within the scope of tolerance zone after the parameters measured through the four measuring methods are compared with their corresponding nominal values, so the measured screw rotor part is qualified after machining. The proposed spot centroid extraction method can improve the LDS measurement accuracy for a free-form surface to a certain degree through a comparison of the measuring results of OMM and LDS 2. The proposed denoising algorithm for point cloud data based on the new-type wavelet threshold function can remarkably improve the processing accuracy of the profile data through a comparison of the measuring results of LDS 1 and LDS 2. The profile accuracy of the screw rotor obtained through the proposed LDS-based on-machine measuring system can reach ±8 μm from the difference values between OMM and CMM results.

In order to guarantee the integrity of the OMM measuring results, it is still necessary to evaluate the measurement uncertainties of major axis *d*_1_, minor axis *d*_2_ and screw pitch *w*. The screw pitch *w* taken as an example, the main factors influencing its measurement uncertainty include: errors of LDS resolution and linearity, *Z*-directional positioning accuracy and guide rail straightness of the machine, environmental temperature change and measurement repeatability. Among them, the Class A evaluation method is used to calculate the measurement uncertainty caused by measurement repeatability, and the measurement uncertainties caused by other factors are calculated through the Class B evaluation method. The influence factors are concretely analyzed as follows.

The LDS resolution can reach 0.1 μm as mentioned in Section 3, namely the measurement uncertainty component introduced by the sensor resolution is *u*_1_ = 0.1 μm. In addition, the linearity error of the LDS is ±0.02% F.S., the nominal value of the screw pitch *w* of the double-headed screw rotor is 102 mm, and this error follows uniform distribution. The coverage factor $k=\sqrt{3}$ is taken, and then the measurement uncertainty component *u*_2_ generated by the LDS linearity error within the measuring range is:

(22) $$u_{2}=\frac{0.02\%F.S.}{k}=\frac{0.02\%\times 102}{\sqrt{3}}=1.18\;\mu m$$

The *Z*-directional positioning accuracy of the LXK100 machine is 0.01 mm/500 mm, and the error it generates follows uniform distribution. $k=\sqrt{3}$ set, the screw length is measured as 110 mm, and then the uncertainty component *u*_3_ induced by the *Z*-directional positioning accuracy of the machine is:

(23) u3=0.01500×1103=0.13 μm

Similarly, the straightness of the axial guide rail of the LXK100 machine is 0.05 mm/1000 mm, and the uncertainty component it causes is *u*_4_ = 0.32 μm.

Under indoor temperature *t* of 9 °C, the OMM method is used to complete the measurement of the screw pitch *w* of the screw rotor 10 times within a short time, and then the measurement uncertainty component *u*_5_ caused by the environmental temperature change is:

(24) $$u_{5}=\alpha_{t}w_{d}\left(t-20\right)$$

where α*_t_* is the linear expansion coefficient of the material, taken as $\alpha_{t}=1.1\times 10^{-6}{/}^{{}^{\circ}}C$; *w_d_* is the nominal screw pitch *w*, namely 102 mm, and then *u*_5_ = −0.12 μm.

The measurement uncertainty *u*_6_ caused by measurement repeatability of the screw pitch *w* is evaluated through the Class A evaluation method, and its standard deviation is calculated through the Bessel equation,

(25) $$s_{x}=\sqrt{\frac{\sum_{i=1}^{n}\left(x_{i}-\bar{x}\right)}{n-1}}$$

where *n* is the number of repeated measurement times; *x_i_* is each measuring result; $\bar{x}$ is the mean of *n* measuring results. The standard deviation of the screw pitch results measured through the OMM method 10 times in Table 3 is calculated as *s_w_*= 0.0012 μm, and the uncertainty it causes is:

(26) $$u_{6}=\frac{s_{w}}{\sqrt{n}}=0.0004\;\mu m$$

As theaforementioned measurement uncertainty components are mutually independent, the OMM can be used to measure the combined uncertainty *u_w_* of the screw pitch *w* of the double-headed screw rotor through the root-sum-square value method as below:

(27) $$u_{w}=\sqrt{u_{1}^{2}+u_{2}^{2}+u_{3}^{2}+u_{4}^{2}+u_{5}^{2}+u_{6}^{2}}=1.24\;\mu m$$

The measurement uncertainties of major axis *d*_1_ and minor axis *d*_2_ are evaluated according to the calculation method for the measurement uncertainty of the screw pitch *w*. In the OMM, the main factors influencing the measurement uncertainties of major axis *d*_1_ and minor axis *d*_2_ include resolution and linearity errors of the LDS, environmental temperature change and measurement repeatability. The standard deviation of major axis *d*_1_ of the double-headed screw rotor in the 10 measurements is calculated as 0.0024 μm and its combined uncertainty as *u_d_*_1_ = 0.72 μm, and the two figures for the minor axis *d*_2_ are 0.0011 μm and *u_d_*_2_ = 0.69 μm, respectively, according to the uncertainty evaluation method of the screw pitch.

To sum up, the measuring accuracy of the proposed LDS-based on-machine measuring system for the double-headed screw rotor can reach as high as ±8 μm, and the measurement uncertainties of major axis, minor axis and screw pitch of the screw rotor are 0.72 μm, 0.69 μm and 1.24 μm, respectively. In the measuring process, the sensor feed speed is *F* = 200 mm/min, the measuring length is 110 mm, and the rotation speed of the principal axis is 10 *r*/min. The measurement of a screw pitch of the screw rotor through the OMM method consumes 39.7 s each time, which can satisfy the requirement for rapid measurement of a large-size workpiece in online production.

## 5. Conclusions

An LDS-based on-machine measuring system for the profile accuracy of a double-headed screw rotor was constructed in this paper. This method measured and rapidly corrected disqualified workpieces in a timely way by taking full advantage of superior CNC resources. In order to realize accurate acquisition of the profile data of double-headed screw rotor, the spot centroid extraction method that could resist noise jamming, a point cloud data denoising algorithm based on a new-type of wavelet threshold function and the point cloud data smoothing method based on the Lagrange multiplier method were proposed, respectively, and their outstanding performances were verified through the simulation and experiment. The proposed data-processing method can be extended to related problems in the fields in which the LDS is used, such as reverse engineering and precision measurement, so it is of a certain guiding significance. The whirlwind milling machines configured with this system and LXK100 four-axis screw rotors have been put into use in the PetroChina Changqing Oilfield. The actual operation results indicate that the measurement accuracy of this method is ±8 μm, in which the measurement uncertainties of major axis, minor axis, and screw pitch are 0.72 μm, 0.69 μm and 1.24 μm, respectively, and measuring one screw pitch consumes 39.7 s. Therefore, the proposed on-machine measuring system can satisfy the requirements for accurate control and rapid measurement of a large workpiece in online production.

## Figures and Tables

**Table 1:** The comparison of results for different threshold functions.

Method	Root-Mean-Square Value (RMS)	Peak Valley (PV)
Grayscale centroid method	0.196	0.387
Gaussian fitting	0.205	0.412
Elliptic fitting	0.183	0.354
Our method	0.147	0.269

**Table 2:** The comparison of results for different threshold functions.

Denoising Method	Signal-to-Noise Ratio (SNR)/dB	Mean Squared Error (MSE)
Hard threshold function	19.577	0.180
Soft threshold function	20.411	0.149
Reference [29]	20.638	0.141
New threshold function	21.226	0.123

**Table 3:** Comparison table of measuring results through different measuring methods.

Method	*d*_1_/mm	*d*_2_/mm	*w*/mm
Coordinate measuring machine (CMM)	59.973	41.983	102.016
Laser displacement sensor 1 (LDS 1)	59.997	41.998	101.986
laser displacement sensor 2 (LDS 2)	59.985	41.991	101.999
On-machine measurement method (OMM)	59.981	41.989	101.008
Error	0.008	0.006	-0.008
Nominal values	$60_{-0.1}^{0}$	$42_{-0.1}^{0}$	$102_{-0.05}^{+0.05}$
